# Decision Making in the Restoration of Endodontically Treated Teeth: Effect of Biomimetic Dentistry Training

**DOI:** 10.3390/dj11070159

**Published:** 2023-06-26

**Authors:** Paridhi Kimble, Sandra Stuhr, Neville McDonald, Akshaya Venugopalan, Marcia S. Campos, Bruno Cavalcanti

**Affiliations:** 1 Department of Cariology, Restorative Sciences and Endodontics, University of Michigan School of Dentistry, Ann Arbor, MI 48109, USA; paridhik@umich.edu (P.K.); somerled@umich.edu (N.M.); akshayav@umich.edu (A.V.); mscampos@umich.edu (M.S.C.); 2 Department of Periodontics and Oral Medicine, University of Michigan School of Dentistry, Ann Arbor, MI 48109, USA; sstuhr@umich.edu

**Keywords:** endodontics, restorative dentistry, biomimetic dentistry, evidence-based dentistry

## Abstract

The restoration of endodontically treated teeth (ETT) is challenging as these teeth often present with structural deficiencies. Currently, there is no consensus regarding the final restoration choice. Historically, the full coverage crown was the universally selected treatment for endodontically treated teeth. With advances in adhesive and biomimetic dentistry, more minimally invasive treatment modalities have become a viable option. With this study, we aim to understand the restorative decision of the general dentist with or without additional training in biomimetic dentistry. Seventy-eight general dentists, with or without biomimetic training, were surveyed to determine their restorative preferences on five extracted posterior teeth, categorized according to volumetric loss of tooth structure, as indicated by the number of missing walls, the isthmus width, the presence or absence of marginal ridges, and cusps. CAD/CAM reconstructions were made with the teeth to analyze the volume of tooth loss and compare these with the survey results. Data were compared using the chi-squared test and Fisher’s exact test. The frequency of responses recommending a crown and the volume of tooth loss were correlated using the Pearson test (*p* < 0.05). For all five teeth, survey responses showed a statistically significant difference in the restorative decision of full coverage versus alternative restorations, with biomimetic dentists selecting a direct restoration or inlay/onlay in lieu of a full coverage crown (*n* = 63, *p* < 0.05). The age of the participant did not have a significant impact on the restorative decision making process for these teeth. The biomimetic trained dentists showed a greater tendency to select a crown option only when the volume of tooth loss was greatest, otherwise their restorative decisions tended towards the conservative treatment options. This study also demonstrates a novel method of digitally developing a volume of tooth loss to compare against the visual interpretation of the volume of tooth loss.

## 1. Introduction

The restoration of endodontically treated teeth (ETT) is a unique challenge for clinicians, as these teeth often present with structural deficiencies due to previous caries, pre-existing restorations, and endodontic access. These biomechanical alterations inflict a negative impact on the long-term prognosis of the tooth [1,2]. Previously, it was believed that clinically and radiographically acceptable root canal filling alone was effective in preventing the ingress of bacteria, thereby promoting the healing of periapical pathosis [3,4,5,6]. Newer studies have challenged this concept by introducing evidence that focuses on the quality of the coronal restoration and its effect on tooth stability. It is now believed that the primary barrier to leakage is not only well-obturated root canals but the seal from the coronal restoration [7,8,9,10]. Corroborating the results from both schools of thought, clinically and radiographically acceptable root canal obturation and coronal restoration remain important goals for the long-term health of the attachment apparatus of teeth [7].

When considering the restoration of endodontically treated teeth, dental materials, ideally, should be able to replace the loss of tooth substance to ensure sufficient mechanical and functional properties, acceptable esthetics, and an adequate coronal seal [11]. Historically, this was fulfilled by full coverage crowns made with fusing porcelain to metal or using porcelain alone. There are several retrospective studies that have supported its use and long-term reliability [12,13], directly associating full coverage restorations with the long term survival and success of root canal treatment [14,15]. Though effective, crowns often require extensive tooth preparation and removal of the natural load bearing areas of the tooth, such as the biorim, that buffer and mitigate tensile forces on the tooth [16,17]. Research on crown failures shows that they often initially fail at the cervical margin, followed by the leakage and subsequent failure of the whole system. The absence of bracing effects of the outer enamel and the dentin enamel complex results in a dependence on the underlying weaker dentin, which is less able to prevent cracks from propagating apically into the root, leading to an unfavorable prognosis [16,18].

Over the past several years, with advances in adhesive dentistry and the introduction of high bonding performances achieved by modern adhesive systems, the clinical recommendation of utilizing a full coverage restoration after endodontic therapy has been questioned [19]. Dentists are universally acquiring additional continuing educational training in the principles of biomimetic dentistry, which focuses on the central idea to “mimic nature” and restore teeth to their original biological structures and biomechanical functions.

The principle of biomimetic dentistry encompasses several factors that guide the treatment decision and the choice of restorative technique. Above all, the preservation of the healthy tooth structure is foundational. By minimizing the removal of the healthy tooth structure, dentists trained in biomimetic dentistry reduce the risk of the weakening of the tooth which would thereby compromise the long-term stability and survival. The preservation of the healthy tooth structure is achieved by incorporating minimally invasive preparation designs that involve the selective removal of caries in the periphery of a vital tooth, often referred to as the peripheral seal zone, which is an area 3 mm from the adjacent tooth and 5 mm occlusally towards the pulp [20]. In the case of an endodontically treated tooth, complete caries removal with the preservation of the remaining cusps is essential, in addition to an access preparation which is often interior to a planned peripheral seal zone. Another principle of biomimetic dentistry involves mimicking the natural biomechanics of the tooth [21]. This is achieved by the careful selection of dental materials and techniques that closely resemble hard dental tissues such as enamel and dentin. By using the natural tooth as an ideal reference, biomimetic restorations are designed to withstand occlusal forces and functional demands that harmonize with the existing tooth structure. This promotes long-term stability, tooth survival, and reduces the risk of failure. 

Bonding and adhesion play a crucial role in biomimetic dentistry. Dentists trained in biomimetic dentistry place a strong emphasis on the use of gold-standard bonding systems rather than relying on mechanical retention. The use of gold standard bonding systems creates a durable bond between the tooth structure and the restoration [22], sealing the margins and preventing bacterial ingress and microleakage. The use of these adhesives enhances the overall strength and longevity of the biomimetic restorations.

Stress reduction is crucial for the long term survival of any restoration [23]. This is achieved through various stress reduction protocols, which includes the incorporation of techniques and materials that minimize C-factor-related stresses [24]. This is accomplished using flowable resin liners, a decoupling with time for the maturation of the adhesive/dentin hybrid layer, the use of ultra-high molecular weight polyethylene fiber, and the use of 1 mm horizontal increments of composites in direct restorations [24,25]. Another technique is to incorporate semi-direct or indirect restorations. This includes the use of inlays, onlays or overlays that are bonded to the tooth while preserving healthy cusps and the peripheral enamel biorim. 

Lastly, dentists trained in biomimetic dentistry are trained to perform a thorough evaluation of the structural compromise of the tooth when making a restorative decision. Factors such as functional and non-functional remaining cusp widths, isthmus width, and depth of proximal boxes are carefully considered [24]. By adhering to the principles of biomimetic dentistry, dentists offer restorations that are conservative, durable, aesthetically pleasing, functionally reliable, and that mimic the biomechanical properties of a natural tooth.

With these newly emerging biomimetic restorative philosophies, the aim of this study was to understand the current perception of general dentists, with and without biomimetics training, and assess factors that guide them in making a restorative decision for endodontically treated teeth. Additionally, the use of digital scanning and 3D modeling software allows for a quantitative analysis of the volume of tooth loss after preparation to compare against a subjective evaluation from each clinician.

## 2. Materials and Methods

For this cross-sectional study, data were collected through an online closed questionnaire conducted through SurveyMonkey. Study participants were selected by participation invitation through the online SurveyMonkey functions. In order to determine an adequate sample size for each group, an independent proportional non-directional two-sided analysis (Pearson chi-Square, *p* < 0.05), with a power of 80%. The minimum sample size for this analysis, including a four-way response, was 22 surveys per group. 

Each participant (general dentists who hold a D.D.S./D.M.D. degree from a CODA-accredited dental school) was shown photographs of five extracted posterior teeth and asked specific questions pertaining to that tooth. The teeth selected had all caries removed using a high-speed handpiece and #330 carbide bur with the aid of caries detector (Kuraray, Houston, TX, USA). All teeth were then accessed with a #1157 carbide bur and all canals were located. Each tooth contained a different volume loss of tooth structure, missing walls, varying isthmus width, and absence of marginal ridges. All teeth were assumed to have supra-gingival margins, Angle’s Class I occlusion with adequate axis, bite, chewing (ABC), and stop-equalizer contacts.

The information gathered through the online survey is summarized in Table 1.

The online hyperlink to the survey was emailed to each participant after explaining the importance of the study. Responses of participants that failed to complete the survey in its entirety were eliminated. 

The five extracted teeth that were used for this survey were scanned using an intraoral scanner (3Shape Trios, Copenhagen, Denmark). A digital restoration replacing only the missing tooth structure was generated for each of the scanned teeth (3Shape Digital Designer, Copenhagen, Denmark) and imported into CAD software (Meshwork, San Diego, CA, USA) to calculate the volume of the restoration alone, and the volume of the clinical crown with the restoration, to determine a percentage of volume of tooth lost using the restoration as an inference.

Data were submitted to descriptive analysis, and associations were made between the restorative decision with age and biomimetic training assessed according to chi-square and Fisher’s exact tests (*p* < 0.05). The frequency of responses recommending a crown and the volume of tooth loss were correlated using the Pearson test (*p* < 0.05).

## 3. Results

A total of 78 dentists were invited to participate in the study and 63 participants (81%) completed the survey (Appendix A Table A1, Table A2, Table A3, Table A4 and Table A5). Out of the 63 participants, 41 (65.08%) were aged 20–40 and 22 (34.92%) participants were aged 41–60. No participants were above the age of 60. Regarding obtaining continuing education in biomimetic dentistry, 28 (44.44 %) participants reported having received more than 12 h of continuing education in biomimetic dentistry and 35 (55.56%) had no formal training in biomimetic dentistry. The responses for the categories for non-cuspal coverage (direct restoration) or partial cuspal coverage (Inlay/onlay/overlay) were analyzed against full cuspal coverage restoration with or without a post. Data were analyzed using a chi-squared test and Fisher’s exact test.

**Figure 1:**
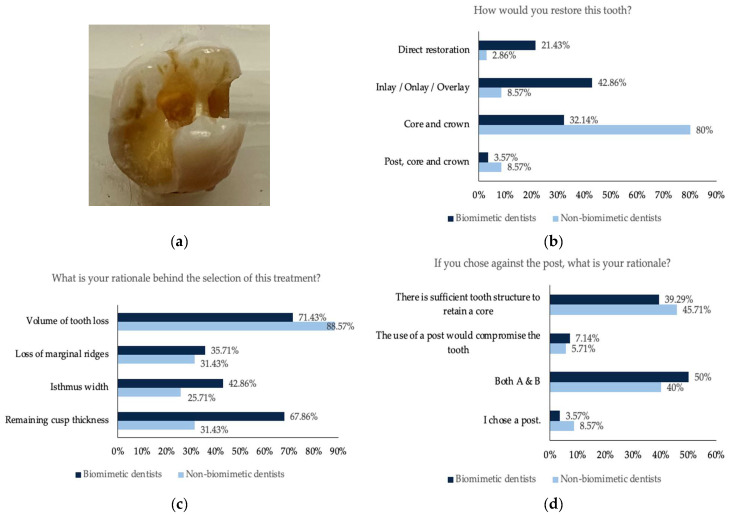
(**a**) Photographs of the maxillary molar tooth with missing mesial and distal marginal ridge and disto-palatal cusp; (**b**) the restorative decision of all the participants obtained from this tooth; (**c**) the rationale behind the choice of the restorative decision; (**d**) the rationale against the use of a post.

**Figure 2:**
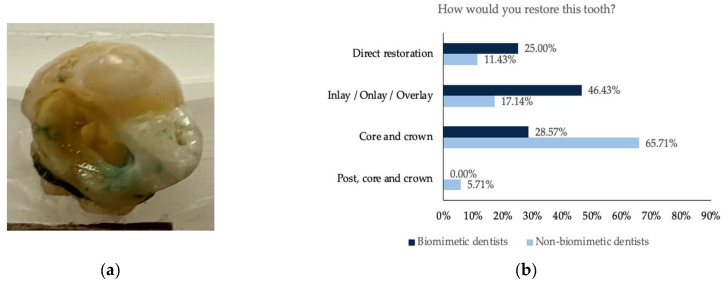

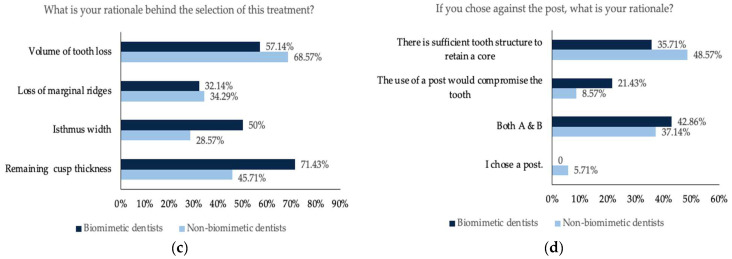
(**a**) Photograph of the maxillary molar tooth with missing distal marginal ridge and disto-palatal and disto-lingual cusp; (**b**) the restorative decision of all the participants obtained from this tooth; (**c**) the rationale behind the choice of the restorative decision; (**d**) the rationale against the use of a post.

**Figure 3:**
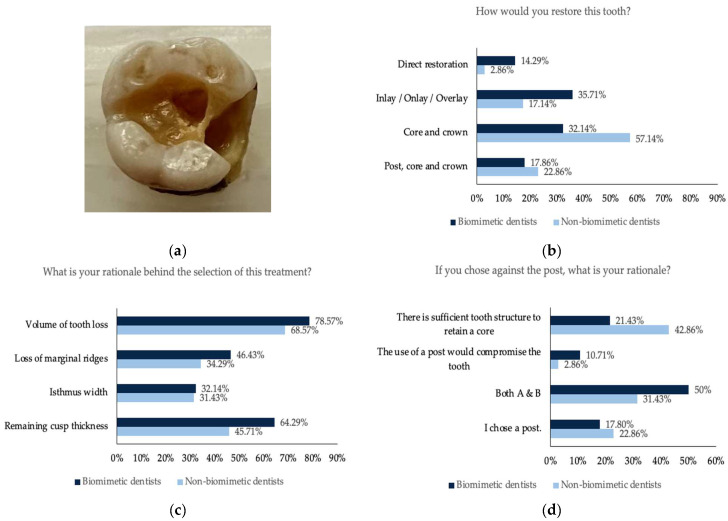
(**a**) Photograph of the maxillary molar tooth with missing mesial marginal ridge; (**b**) the restorative decision of all the participants obtained from this tooth; (**c**) the rationale behind the choice of the restorative decision; (**d**) the rationale against the use of a post.

**Figure 4:**
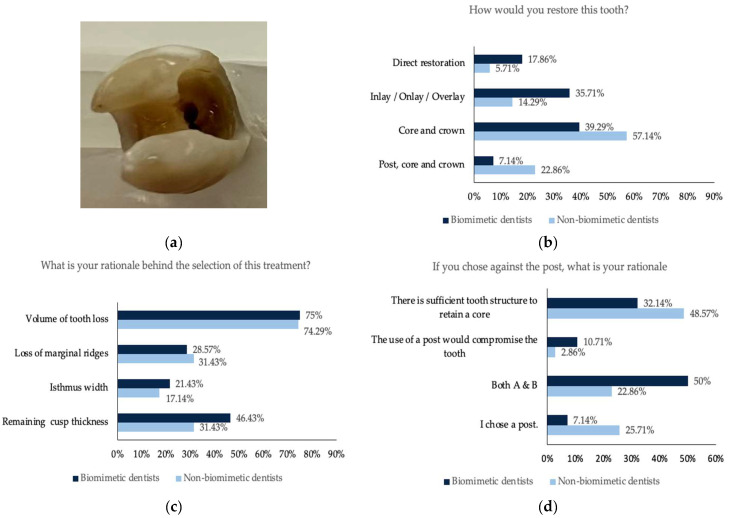
(**a**) Photographs of the maxillary premolar tooth missing mesial and distal marginal ridge; (**b**) the restorative decision of all the participants obtained from this tooth; (**c**) the rationale behind the choice of the restorative decision; (**d**) the rationale against the use of a post.

**Figure 5:**
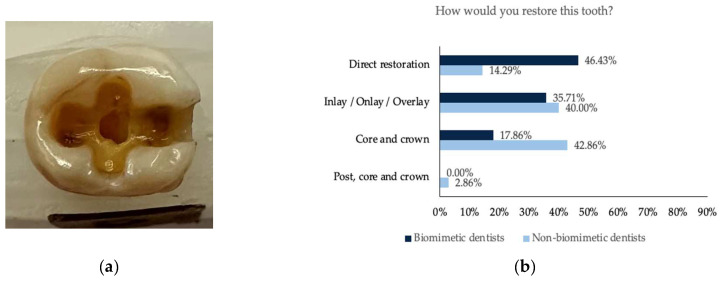

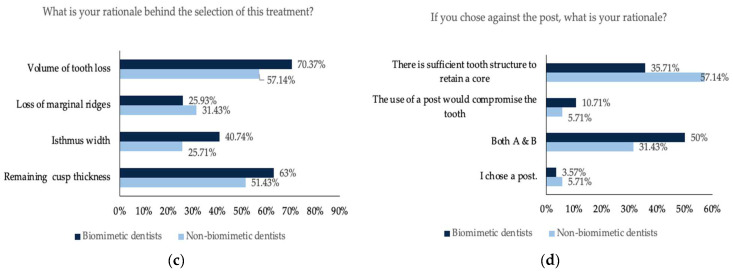
(**a**) Photograph of the mandibular molar with missing mesial marginal ridge; (**b**) the restorative decision of all the participants obtained from this tooth; (**c**) the rationale behind the choice of the restorative decision; (**d**) the rationale against the use of a post.

For all five teeth (Figure 1, Figure 2, Figure 3, Figure 4 and Figure 5), survey responses showed a statistically significant difference in the restorative decision of full coverage versus alternative restorations, with dentists trained in biomimetic dentistry selecting a direct restoration or inlay/onlay in lieu of a full coverage crown (*n* = 63, *p* < 0.05). The age of the participant did not have a significant impact on the restorative decision making process for these teeth. There was no significant difference between biomimetic training and the choice of placement of a post (*n* = 63, *p* > 0.05). For all respondents across all teeth, the volume of tooth loss was the most selected rationale for their restorative decision, irrespective of their biomimetic training and age. With regards to the use of a post, both dentists with or without training in biomimetic dentistry did not opt for a post, though the rationale selected by the dentists trained in biomimetic dentistry was that the use of a post would be detrimental to the tooth, whereas dentists not trained in biomimetic dentistry selected that there was sufficient tooth structure.

**Figure 6:**
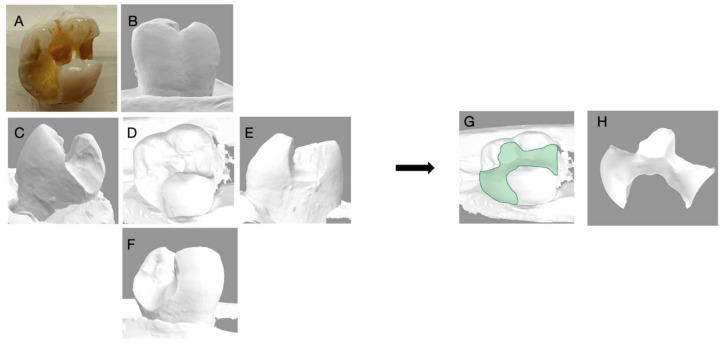
Depicts the 3D reconstruction of the maxillary molar acquired by 3Shape Digital Designer, (3Shape Copenhagen, Denmark). (**A**) Photograph of the extracted tooth; (**B**) the buccal view; (**C**) The distal view; (**D**) the occlusal view; (**E**) the mesial view; (**F**) the lingual view of the 3D scan; (**G**) the 3D reconstruction with an inlay; (**H**) the inlay proposal of the tooth generated with 3D shaped digital designer.

**Figure 7:**
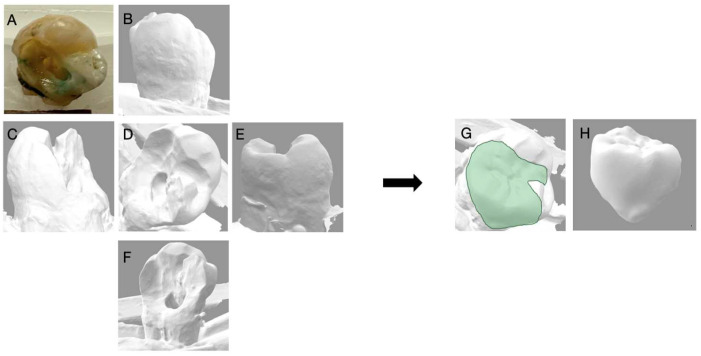
Depicts the 3D reconstruction of the maxillary molar acquired by 3Shape Digital Designer, (3Shape Copenhagen, Denmark). (**A**) Photograph of the extracted tooth; (**B**) the buccal view; (**C**) the distal view; (**D**) the occlusal view; (**E**) the mesial view; (**F**) the lingual view of the 3D scan; (**G**) the 3D reconstruction with an inlay; (**H**) the inlay proposal of the tooth generated with 3D shaped digital designer.

**Figure 8:**
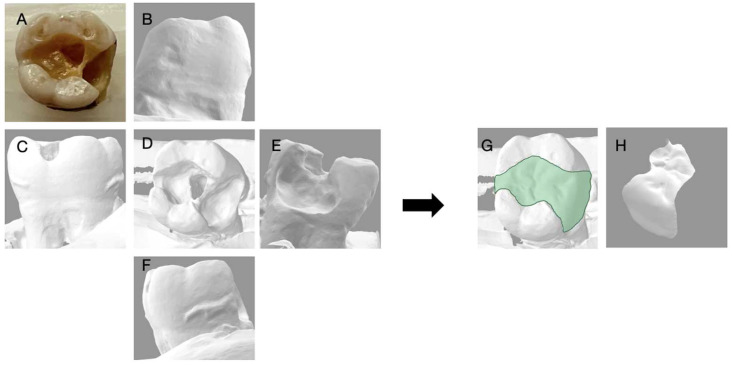
Depicts the 3D reconstruction of the maxillary molar acquired by 3Shape Digital Designer, (3Shape Copenhagen, Denmark). (**A**) Photograph of the extracted tooth; (**B**) the buccal view; (**C**) the distal view; (**D**) the occlusal view; (**E**) the mesial view; (**F**) the lingual view of the 3D scan; (**G**) the 3D reconstruction with an inlay; (**H**) the inlay proposal of the tooth generated with 3D shaped digital designer.

**Figure 9:**
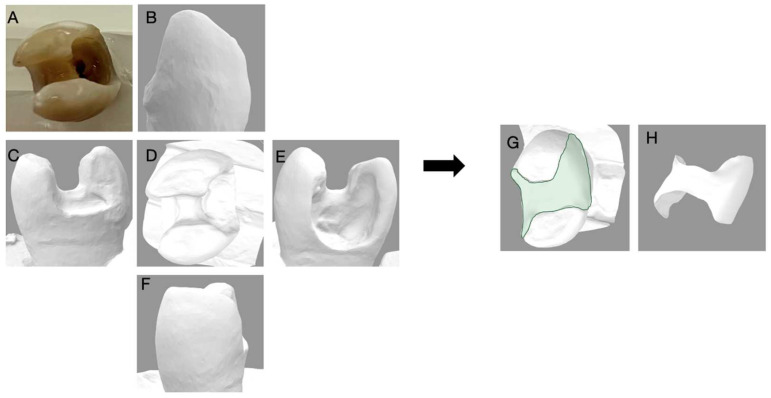
Depicts the 3D reconstruction of the maxillary premolar acquired by 3Shape Digital Designer, (3Shape Copenhagen, Denmark). (**A**) Photograph of the extracted tooth; (**B**) the buccal view; (**C**) the distal view; (**D**) the occlusal view; (**E**) the mesial view; (**F**) the lingual view of the 3D scan; (**G**) the 3D reconstruction with an inlay; (**H**) the inlay proposal of the tooth generated with 3D shaped digital designer.

**Figure 10:**
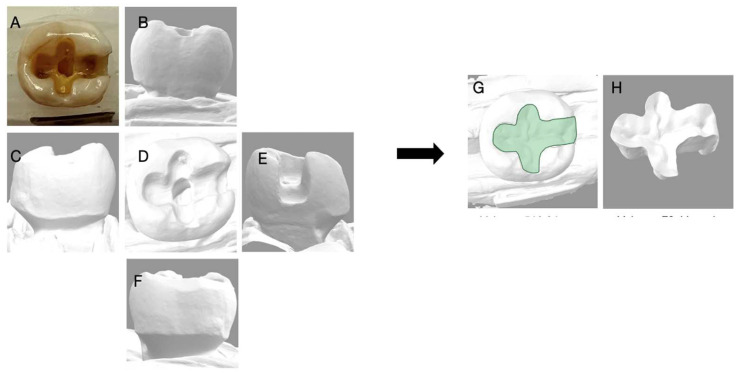
Depicts the 3D reconstruction of the mandibular molar acquired by 3Shape Digital Designer, (3Shape Copenhagen, Denmark). (**A**) Photograph of the extracted tooth; (**B**) the buccal view; (**C**) the distal view; (**D**) the occlusal view; (**E**) the mesial view; (**F**) the lingual view of the 3D scan; (**G**) the 3D reconstruction with an inlay; (**H**) the inlay proposal of the tooth generated with 3D shaped digital designer.

**Figure 11:**
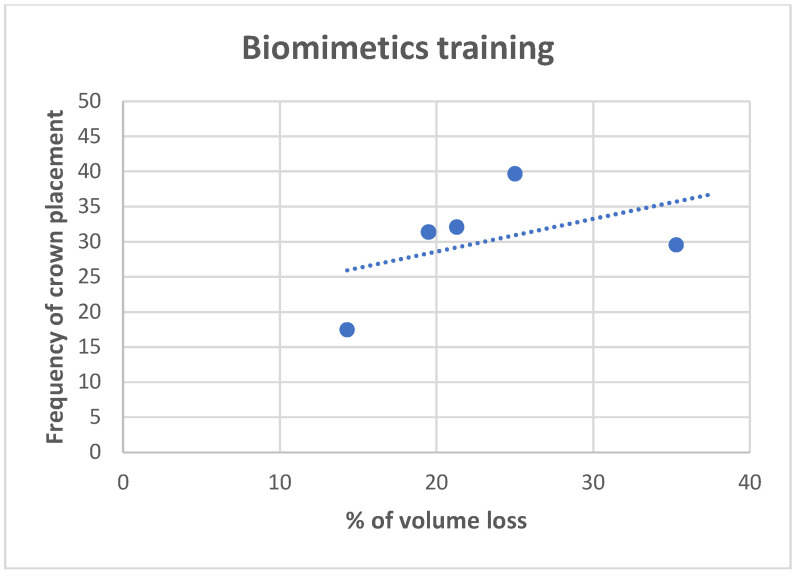
Correlation analysis for the volume of tooth loss, and frequency of decision for full crown restoration, for dentists trained in biomimetic dentistry. The Pearson correlation value is 0.4563, indicating moderate positive correlation.

**Figure 12:**
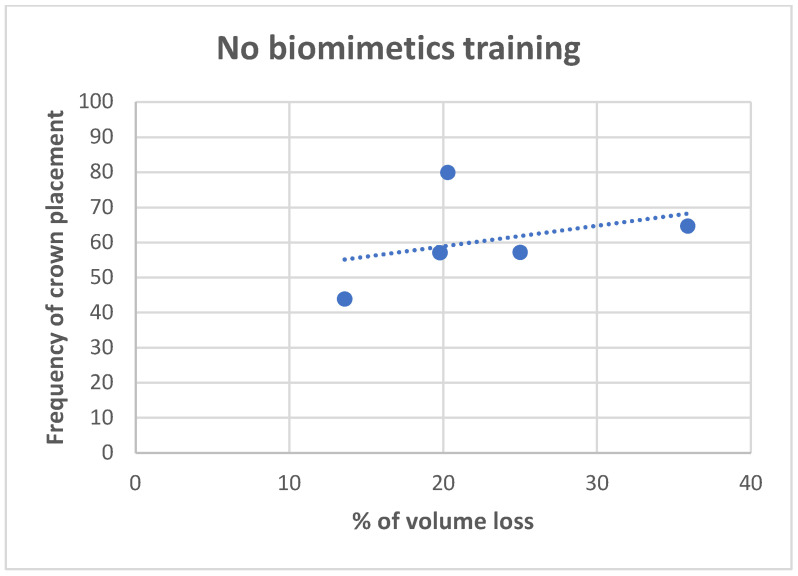
Correlation analysis for the volume of tooth loss, and frequency of decision for full crown restoration, for dentists not trained in biomimetic dentistry. The Pearson correlation value is 0.3691, indicating weak correlation.

The volume of tooth loss data are summarized in Table 2 and Figure 6, Figure 7, Figure 8, Figure 9 and Figure 10. Regarding the correlation between the volume of tooth loss and the decision to place a crown, it was observed that there is a moderate positive correlation for dentists trained in biomimetic dentistry (R = 0.4563, while only a weak correlation was found for the dentists not trained in biomimetic dentistry (R = 0.3691). The correlation charts can be observed, respectively, in Figure 11 and Figure 12.

## 4. Discussion

This study aimed to investigate the restorative decision making process of dentists for endodontically treated teeth, with particular focus on individual backgrounds in biomimetic dentistry and the impact of the dentist’s age range. The key findings of the study were that dentists trained in biomimetic dentistry were more likely to select a direct restoration or inlay/onlay in lieu of a full coverage crown compared to dentists not trained in biomimetic dentistry. The difference in decision making was statistically significant across all five teeth analyzed, indicating that biomimetic dentistry training had a significant impact on the restorative decision making process.

These findings are consistent with the biomimetic theory of conservative restorative decisions that often maximize the remaining tooth structure and minimize further preparation. In cases of endodontically treated teeth, the biomimetic strategy would avoid preparing a post space and performing a 360-degree preparation, and select for strengthening the remaining dentin-resin bond and prevent further stress and strain within the restoration [26]. Bonding is preferred over mechanical retention, suggesting why dentists often select the use of adhesive and fiber reinforcement in an effort to mimic the dental hard tissues that are being restored [27]. In contrast, a practitioner that does not perform biomimetic techniques may rely on the reliability of a full coverage crown. In a retrospective study by Pratt et al. [28], a typical composite/amalgam buildup was observed to have a 2.29 greater likelihood of extraction in comparison to a full coverage crown, 11.6% and 5.7%, respectively.

Another consideration for a biomimetic approach to preserving the remaining tooth structure, in lieu of full coverage, is the longevity of the tooth, not necessarily the restoration. While there is a concern that an inlay or remaining tooth could fracture, this may be converted into another restoration in the future, depending on the fracture location. The failure of a conservative restoration may not necessarily result in the failure of the tooth; however, a fully prepared crown may not be as amenable to further restorative treatments, even though the annual failure rates among these restorations have been reported to be similar [29]. Our study adds to the existing literature by providing insights on the differences in the restorative decision making process, specifically in endodontically treated teeth in order to promote evidence-based decision making.

With regards to biomechanical factors, among the responses, the volume of tooth loss seemed to be the most important factor in a restorative decision, yet we see varied restorative methods based on biomimetic training. One could infer that dentists trained in biomimetic dentistry are maximizing the restorative prognosis for a tooth without reducing the tooth volume. In other words, dentists not trained in biomimetic dentistry seek to restore a tooth with a full-coverage crown due to the volume of tooth loss, which, by necessity, reduces the volume further. However, dentists trained in biomimetic trained dentistry may see the volume of tooth loss as antithetical to a restorative goal and seeks to restore with as little reduction as possible. I In a study by Larson [30], the influence of surface preparation is highly correlated with fracture risk. While the total volume of tooth loss may be a subjective evaluation of the tooth holistically, marginal ridge loss, isthmus width [31], and remaining cusp thickness are well-understood biomechanical properties of fracture resistance [32]. In our study, we also observed that it is possible that the biomimetic training has more influence in correlating a higher loss of tooth structure with the indication of a crown, while dentists without training, although using the volume of tooth loss as justification, do not correlate the actual volume of tooth loss with the indications for crowns, which were more prevalent independently of the study tooth.

It is important to acknowledge the limitations of this study. As with any survey, there is always a limited sample size, making it difficult to stratify by all restorative methods. Categories of direct and inlay/onlay, for example, were combined against core and crown and post core and crown, in order to be analyzed in a Fischer exact test. However, based on our power analysis, we were able to detect the major differences between the biomimetics trained and non-trained groups, providing a good and statistically sound insight into how training affects clinical thought processes and, thus, dentists’ decisions. The use of extracted teeth that are prepared and photographed may not be comparable to an in situ tooth presented to a clinician in a practice setting. The digital evaluation for the volume of tooth structure is a novel method of quantifying what, traditionally, has been a subjective evaluation by a dental practitioner. In our study, we used a volume of tooth loss ranging from 13.5 to 35.9%, which showed no correlation with the choice of a full cusp coverage restoration by a dentist not trained in biomimetic dentistry. A future study could include larger volumes of tooth reduction (>50.0%) to evaluate if a correlation exists with larger volume loss. In our study, we included only restorative factors such an isthmus width, remaining cusp thickness, and loss of marginal ridges. In practice settings, there are other factors, such as patient disposition, finances, dental history, occlusion, periodontal status of the tooth and presence or absence of adjacent and contralateral teeth, that were not included that could influence a dentist’s restorative decision. While no results were significant when stratified by age groups, there could be a bias that was unaccounted for. Biomimetic training is a relatively newer restorative trend which may not be readily practiced or understood by more practiced dentists, yet newly practicing dentists may also not have had comprehensive training in partial coverage indirect therapies, such as inlays or onlays, which can be more technique-sensitive; the practice of full coverage restorations is an anthem for many more newly trained dentists. The findings of this study have important implications for clinical activity, particularly for dentists who are considering incorporating biomimetic techniques into their practice. This study suggests that biomimetic training can lead to more conservative and tooth-preserving restorative decisions, which may have long-term benefits for patients in terms of tooth function and longevity. Currently, the evidence to support the longevity of biomimetic restorations is limited. There is a need for prospective long-term studies that follow biomimetic restorations to evaluate survival and to compare with other restorative outcomes. As discussed before, the longevity of the restoration is not synonymous with the longevity of the tooth, and preserving tooth structure can consequently result in tooth longevity, even if the restoration itself may fail. 

It is important for dentists to consider their own experience and skill level when implementing biomimetic techniques and to carefully weigh the potential benefits and risks of each treatment option for each individual patient. Moreover, the literature needs more studies on the long-term clinical outcomes of these conservative techniques when dealing with endodontically treated teeth.

## 5. Conclusions

Within the limitations of this study, it can be concluded that the restorative decision for endodontically treated teeth, made by a dentist trained in biomimetic dentistry, is significantly different than a dentist not trained in biomimetic dentistry. There is a direct correlation between the volume of tooth loss with the recommendation of a full coverage crown for dentists trained in biomimetic dentistry and no correlation with the volume of tooth loss with dentists not trained in biomimetic dentistry. The novel utilization of calculating the volume of tooth loss using digital modeling, as a method to derive and compare a quantifiable volume against a visual interpretation of a volume of tooth loss, could be used in the future to calibrate restorative decisions. Based on the survey results, it can be concluded that dentists trained in biomimetic dentistry can be expected to make more conservative restorative decisions, consistent with their training, than dentists not trained in biomimetic dentistry. 

## Figures and Tables

**Table 1:** Overview of the online survey.

Question	Responses
1. How would you restore this tooth?	A. Direct restoration B. Inlay/Onlay/Overlay C. Core and crown D. Post, core, and crown
2. What is your rationale for the selection of this treatment?	A. Volume of tooth loss B. Loss of marginal ridges C. Isthmus width D. Remaining cusp thickness
3. If you chose against the use of a post, what is your rationale?	A. There is sufficient tooth structure to retain a core B. The use of a post would compromise the tooth C. Both A & B D. I chose a post.
4. How old are you?	A. 20–40 B. 41–60
5. Have you received 12 or more hours of continued education in biomimetic Dentistry?	A. Yes B. No

**Table 2:** Summary of volume of tooth loss.

Tooth	Volume (mm^3^)		Percentage Loss (%)
	Scanned Tooth	Digital Restoration	
1	584.49	124.47	21.3
2	485.85	174.72	35.9
3	491.85	97.54	19.8
4	225.82	57.99	25.0
5	516.24	70.41	13.6

## Data Availability

The full dataset is available as an appendix to this manuscript.

## Appendix A

**Table A1:** Responses for tooth 1 grouped by biomimetic training and age.

Responses		Biomimetic Training N (%)		Age N (%)	
		Biomimetic Dentist	Non Biomimetic Dentist	20–40	41–60
1	Direct	6 (21.3)	1 (2.7)	3 (7.2)	4 (18.2)
	Inlay and Onlay	12 (42.9)	3 (8.6)	12 (29.3)	3 (13.6)
	Core and crown	9 (32.1)	28 (80.0)	25 (60.9)	12 (54.5)
	Post, core, and crown	1 (3.6)	3 (8.6%)	1 (2.4)	3 (13.6)
2	Volume of tooth loss	20 (71.4)	31 (88.6)	34 (82.9)	17 (77.3)
	Loss of marginal ridge	10 (35.7)	11 (31.4)	15 (36.6)	6 (27.3)
	Isthmus width	12 (42.9)	9 (25.7)	16 (39.0)	5 (22.8)
	Cusp thickness	19 (67.9)	11 (31.4)	20 (48.7)	10 (45.4)
3	Sufficient tooth structure	11 (39.3)	16 (45.7)	19 (46.3)	8 (36.4)
	Comprises the tooth	2 (7.2)	2 (5.7)	4 (9.8)	0 (0.0)
	Both A and B	14 (50.0)	14 (40.0)	17 (41.5)	11 (50.0)
	I chose a post	1 (3.6)	3 (8.6)	1 (2.4)	3 (13.6)

**Table A2:** Responses for tooth 2 grouped by biomimetic training and age.

Responses		Biomimetic Training N (%)		Age N (%)	
		Biomimetic Dentist	Non Biomimetic Dentist	20–40	41–60
1	Direct	7 (25.0)	4 (11.4)	5 (12.2)	6 (27.3)
	Inlay and Onlay	13 (46.4)	6 (17.1)	14 (34.1)	5 (22.7)
	Core and crown	8 (28.6)	23 (65.7)	21 (51.2)	10 (45.4)
	Post, core, and crown	0 (0.0)	2 (5.7)	1 (2.4)	1 (4.5)
2	Volume of tooth loss	16 (57.1)	24 (68.6)	30 (73.2)	10 (45.4)
	Loss of marginal ridge	9 (32.1)	12 (34.3)	15 (36.6)	6 (27.3)
	Isthmus width	14 (50.0)	10 (28.6)	19 (46.3)	5 (22.7)
	Cusp thickness	20 (71.4)	16 (45.7)	22 (53.7)	14 (63.6)
3	Sufficient tooth structure	10 (35.7)	17 (48.6)	19 (46.3)	8 (36.4)
	Comprises the tooth	6 (21.4)	3 (8.6)	6 (14.63)	3 (13.6)
	Both A and B	12 (42.7)	13 (37.1)	15 (36.6)	10 (45.4)
	I chose a post	0 (0.0)	2 (5.7)	1 (2.4)	1 (4.5)

**Table A3:** Responses for tooth 3 grouped by biomimetic training and age.

Responses		Biomimetic Training N (%)		Age N (%)	
		Biomimetic Dentist	Non Biomimetic Dentist	20–40	41–60
1	Direct	4 (14.3)	1 (2.9)	2 (4.9)	3 (13.6)
	Inlay and Onlay	10 (35.7)	6 (17.1)	10 (24.4)	6 (27.2)
	Core and crown	9 (32.1)	20 (57.1)	20 (48.8)	9 (40.9)
	Post, core, and crown	5 (17.9)	8 (22.9)	9 (21.9)	4 (18.2)
2	Volume of tooth loss	22 (78.6)	24 (68.6)	32 (78.0)	14 (63.6)
	Loss of marginal ridge	13 (46.4)	12 (34.3)	18 (43.9)	7 (31.8)
	Isthmus width	9 (32.1)	11 (31.4)	15 (36.6)	5 (22.7)
	Cusp thickness	18 (64.3)	16 (45.7)	23 (56.1)	11 (50.0)
3	Sufficient tooth structure	6 (21.4)	15 (42.9)	13 (31.7)	8 (36.4)
	Comprises the tooth	3 (10.7)	1 (2.9)	3 (7.3)	1 (4.5)
	Both A and B	14 (50.0)	11 (31.4)	16 (39.0)	9 (40.0)
	I chose a post	5 (17.9)	8 (22.7)	9 (21.9)	4 (18.2)

**Table A4:** Responses for tooth 4 grouped by biomimetic training and age.

Responses		Biomimetic Training N (%)		Age N (%)	
		Biomimetic Dentist	Non Biomimetic Dentist	20–40	41–60
1	Direct	5 (17.9)	2 (5.7)	5 (12.2)	2 (9.1)
	Inlay and Onlay	10 (35.7)	5 (14.3)	10 (24.4)	5 (22.7)
	Core and crown	11 (39.3)	20 (57.2)	19 (46.3)	12 (54.5)
	Post, core, and crown	2 (7.1)	8 (22.9)	7 (17.1)	3 (13.6)
2	Volume of tooth loss	21 (75.0)	26 (74.3)	32 (78.1)	15 (68.2)
	Loss of marginal ridge	8 (28.6)	11 (31.4)	14 (34.1)	5 (22.7)
	Isthmus width	6 (21.4)	6 (17.1)	8 (19.5)	4 (18.2)
	Cusp thickness	13 (46.4)	11 (31.0)	13 (31.7)	11 (50.0)
3	Sufficient tooth structure	9 (32.1)	17 (48.6)	15 (36.6)	11 (50.0)
	Comprises the tooth	3 (10.7)	1 (2.7)	3 (7.3)	1 (4.5)
	Both A and B	14 (50.0)	8 (22.7)	16 (39.1)	6 (27.3)
	I chose a post	2 (7.1)	9 (25.7)	7 (17.1)	4 (18.2)

**Table A5:** Responses for tooth 5 grouped by biomimetic training and age.

Responses		Biomimetic Training N (%)		Age N (%)	
		Biomimetic Dentist	Non Biomimetic Dentist	20–40	41–60
1	Direct	13 (46.4)	5 (14.3)	14 (34.1)	4 (18.2)
	Inlay and Onlay	10 (35.7)	14 (40.0)	15 (36.6)	9 (40.9)
	Core and crown	5 (17.9)	15 (42.9)	12 (29.3)	8 (36.4)
	Post, core, and crown	0 (0.0)	1 (2.9)	0 (0.0)	1 (4.5)
2	Volume of tooth loss	19 (70.4)	20 (57.1)	26 (65.0)	13 (59.1)
	Loss of marginal ridge	7 (25.9)	11 (31.4)	13 (32.5)	5 (22.7)
	Isthmus width	11 (40.7)	9 (25.7)	14 (35.0)	6 (27.3)
	Cusp thickness	17 (62.9)	18 (51.4)	22 (55.0)	13 (59.1)
3	Sufficient tooth structure	10 (35.7)	20 (57.1)	19 (46.3)	11 (50.0)
	Comprises the tooth	3 (10.7)	2 (5.7)	2 (4.9)	3 (13.6)
	Both A and B	14 (50.0)	11 (31.4)	18 (43.9)	7 (31.8)
	I chose a post	1 (3.6)	2 (5.7)	2 (4.8)	1 (4.5)
